# Knowledge, Perception and Consumption of Indigenous Foods in Gauteng Region, South Africa

**DOI:** 10.3390/ijerph20206961

**Published:** 2023-10-23

**Authors:** Hema Kesa, Alex D. Tchuenchieu Kamgain, Mthokozisi Kwazi Zuma, Xikombiso Mbhenyane

**Affiliations:** 1 Food Evolution Research Laboratory, School of Tourism and Hospitality, University of Johannesburg, Johannesburg 2092, South Africa; 2 Division of Human Nutrition, Department of Global Health, Faculty of Medicine and Health Science, Stellenbosch University, Cape Town 8000, South Africa; 3 Centre for Food, Food Security and Nutrition Research, Institute of Medical Research and Medicinal Plants Studies, Yaoundé 13033, Cameroon; 4 Agricultural Research Council, Central Office, Smallholder Agricultural Development Unit, Pretoria 0002, South Africa

**Keywords:** diet-related diseases, public health, indigenous foods, consumption, Gauteng Province

## Abstract

Urbanisation in South Africa has led to a nutritional transition from traditional diets (mainly based on indigenous foods) to a Western diet. Currently, the country is one of the most concerned about the prevalence of associated malnutrition and non-communicable diseases. One should, therefore, question the position of indigenous foods (IFs) in the population’s eating habits since their nutritional and health value is known. This study aimed to collect updated data on South Africans’ true awareness and consumption of indigenous foods, especially in the Gauteng region (the most urbanised province of the country). A quantitative cross-sectional research survey was conducted (n = 746). Among a list of 18 IFs, grain sorghum 32.4% (n = 242), marula 32% (n = 239), pearl millet 21.7% (n = 162), amadumbe 19.3% (n = 144) and cowpea 18.6% (n = 139) were the best known. However, the study noticed a maximum consumption of 19.3% (grain sorghum). Overall, this consumption was seasonal, and its level was significantly defined by race (*p* < 0.05). Black people consume more IFs compared to coloured people, Indian people and white people. Participants mostly consumed these foods for nutritional and health reasons and pointed out the problem of availability. “Poor image” was rated the lowest by all races (black 5.8%, coloured 4.2%, Indian 7.0% and white 4.1%) regarding the reasons for no or low consumption of IFs. Whatever the race, the desire to increase IF consumption was positive. The promotion of their integration into South African diets should, therefore, be considered as an intervention strategy.

## 1. Introduction

Over the last decade, concern has been mounting in the Global South over the rapid rise in the prevalence of non-communicable diseases (NCDs) and the health and economic burden that they represent [1,2,3,4,5]. NCDs (mainly cardiovascular disease, cancer, diabetes and chronic respiratory disorders) have become a critical issue for low- and middle-income countries (LMICs) and are mainly caused by four behavioural risk factors: diet, physical activity, smoking and alcohol. In 2018, the World Health Organization (WHO) reported that 71% of deaths worldwide are NCD-related, of which 78% occur in LMICs, and about 29% of these deaths are people under the age of 60 [6]. Once affected by NCDs, people often live with the consequences for the rest of their lives. Food, diet and nutritional status are important determinants of NCDs [7,8]. Poor eating habits contribute to the NCD problem more than any other risk factors, like tobacco, alcohol abuse and physical inactivity [9].

Worldwide, populations are increasingly exposed to foods and diets that influence the risk of developing NCDs. Calories obtained from meat, sugars, oils and fats have been increasing globally during recent decades, while calories obtained from fibre-rich foods, such as whole grains, pulses, roots, fruits, vegetables and other indigenous foods, have been decreasing. The consumption of processed and convenience foods continues to rise rapidly in LMICs. Vorster et al. [10] state that modernisation and economic development are associated with nutrition transition. These changes include a decreased intake of staple foods that are rich in starch and dietary fibre, increased consumption of food from animal origin (which is rich in total and saturated fat), decreased intake of legumes and vegetables and increased intake of energy-dense, micronutrient-poor snacks, convenience foods (which are often very salty) and sweetened carbonated beverages. Although more fruit consumption was observed, the increased meat and fruit intake was insufficient to meet micronutrient needs [10]. This nutrition transition affects dietary patterns and nutrient intake, which influence the risk of developing NCDs [11,12].

At present, the consumption of indigenous foods (IFs) is promoted to reduce diet-related disease prevalence. They represent foods that originate in a region, are culturally acceptable, adapted to local climatic conditions and have been consumed traditionally by the inhabitants as opposed to exotic foods that have been introduced from other regions of the world [13]. Indigenous foods are rich and inexpensive sources of proteins, carbohydrates, dietary fibre, minerals and vitamins for millions of people in developed and LMIC countries and are some of the staple foods of the indigenous populations of Africa [11,14,15,16]. Furthermore, the key advantages of these indigenous foods are their adaptability to adverse environmental conditions, resistance to pests, cultural acceptability and sufficient nutritional qualities [13].

In South Africa, traditional diets are being replaced by energy-dense, processed foods, mainly driven by the food industry. Indigenous foods have long been the main source of nourishment for many South Africans. These are crops originating in South Africa, as well as crops that have been introduced into the country and are now considered traditional [17]. The traditional diet was predominantly based on staple grains or starchy roots, legumes, leafy vegetables and fruits and minimal animal products. The production and consumption of nonindigenous foods, commonly known as a Western diet, introduced foods that have led to the displacement of indigenous food, causing a significant change in the diets of many South Africans. The reduction in the consumption of indigenous foods, accompanied by changes in dietary intake, places people (especially those in rural areas) at risk of malnutrition and other nutrition-related or non-communicable diseases such as cardiovascular diseases and diabetes [18]. South Africa has a high rate of malnutrition and undernutrition, and NCDs are among the leading causes of death [19]. According to a 2018 WHO report, NCDs accounted for 51% of total deaths in South Africa in 2016, especially cardiovascular diseases, cancers, diabetes and chronic respiratory diseases, with diet being the most important behavioural risk factor [11]. This contrasts with the diversity of indigenous South African food crops, which are known and treasured for their use in treating or preventing diseases beyond their basic function of the supply of nutrients and energy. Knowledge of these special foods was carefully passed down from generation to generation [11,20,21] and might vary between races, similar to eating habits. This study aimed to collect updated data on the real awareness and consumption of indigenous foods by South Africans, which could aid in combatting the problem of malnutrition and NCDs, with the target area for the study being the Gauteng Province (the most urbanised region of the country).

## 2. Materials and Methods

### 2.1. Study Design

The study followed a descriptive cross-sectional quantitative design.

### 2.2. Study Site and Population

The Gauteng region was selected since there are limited data on the consumption patterns of indigenous foods by the population of this province. It is also representative of most racial and cultural groups in South Africa. Gauteng is home to 15.7 million people (2019 StatsSA mid-year estimate), which is 25.8% of the total South African population. The province’s age distribution at that time was 23.6% below the age of 15, 19.6% between 15 and 24, 37.9% from 25 to 44, 15.0% from 45 to 64 and 4.0% 65 years or older. The median age is 27 years. In the province, the languages spoken at home by residents are: 19.5% IsiZulu, 14.4% Afrikaans, 13.1% Sesotho, 12.5% English, 11.4% Setswana, 10.7% Sepedi, 6.3% IsiXhosa, 4.1% Xitsonga, 3.2% Tshivenda, 1.5% IsiNdebele and 1.2% SiSwati. About 2.0% of the population speaks an unofficial language at home (Stats SA, 2011).

The survey was conducted from August to November 2019 in the metropolitan municipalities of the Gauteng province (City of Ekurhuleni, City of Johannesburg and City of Tshwane Metropolitan Municipalities) with the inclusive criteria of people aged above 18 years old and living in the province for at least two years.

### 2.3. Sampling

The study employed random sampling, which is a process whereby each member of the subset of a population has an equal probability of being chosen [22,23]. The participants were randomly approached in public areas from the selected areas using a systematic approach of asking every fourth person encountered if they were available and willing to participate in the research.

The size was calculated from 15.7 million minus 23.6% of children under 18 years by using Slovin’s formula:

n = N/(1 + Ne^2^) people (1)

n = 11,994,800/(1 + 11,994,800 × 0.0025)

n = 11,994,800/29,988

n = 399.99

Thus, the sample size was 400 plus 10% to accommodate attrition, accruing to 440.

Once the analysis had been completed and the data cleaned, the study used the true sample size of 746. The reason for using the actual sample size was to ensure a comprehensive representation of the population in the different areas of Gauteng. During data collection, some areas of Gauteng, like the City of Johannesburg Metropolitan Municipality, comprised a larger area (including urban and peri-urban areas), consisting of 46% of the study population and higher than the planned sample size. Therefore, the actual sample size was included.

### 2.4. Data Collection

The survey was conducted using a closed-ended, self-administered questionnaire developed to collect data on the sociodemographic characteristics of the respondents, their knowledge and consumption of each of the official common South African indigenous foods [17], their frequency of consumption, their perceptions and the reasons why they use those indigenous crops. The sociodemographic questions focused on the participants’ gender, race, age, education level, household size and income per month, area of residence and corresponding settlement. For each of the listed IFs, respondents had to select “yes” or “no” for knowledge and “yes” or “no” for consumption, and had to inform about their consumption frequency (daily, weekly, monthly, seasonally or rarely) for those foods consumed. Participants also had to inform about their willingness to increase IF consumption (“yes”, “maybe” or “no”). The questionnaire posed multiple-choice questions about the participants’ motivation for consuming IF and reasons for non or low consumption of IF.

The survey was executed with the help of field workers trained in obtaining informed consent and administering questionnaires. The data were collected from public areas (malls, churches and community centres) in various municipalities or metros in Gauteng. The aims of the study were explained to the participants. They were also informed that the information would remain confidential and anonymous, and that their details were not recorded on the questionnaires.

The questionnaire was in English. The researchers launched a pilot study with a diverse group of participants consisting of 15 people from different areas in Gauteng to assess the validity and reliability of the questionnaire. Those individuals were not included in the final dataset. No changes were made to the tools after the pilot study had been conducted. The field workers recruited for the study were able to communicate in all the official languages of South Africa. This assisted in ensuring that the surveys were correctly completed.

### 2.5. Ethical Considerations

This study received approval from the Research Ethics Committee of the University of Johannesburg, ethics clearance number 2019STH012 (10 April 2019) and a waiver from Stellenbosch University, HREC, ethics clearance number X20/11/040 (17 November 2020). Informed consent was obtained from each participant after the research objectives had been explained. Privacy and confidentiality were thoroughly maintained. The completed questionnaires were secured in a locked room. There were no physical risks to participants since there were no interventions, e.g., blood sampling during the study.

### 2.6. Data Analysis

The collected data were entered into *Excel* and then imported into and analysed with the *Statistical Package for Social Sciences* (*SPSS*) version 27 (IBM *SPSS Statistics*, Chicago, IL, USA) for descriptive analysis (frequencies and percentages). The study utilised cross-tabulation and chi-square tests to determine the association between race and the consumers’ knowledge of indigenous foods. A multinomial logistic regression analysis was performed to estimate the association between race and consumption of indigenous crops, considering the consumption of indigenous crops as the outcome and race as the independent variable. White people were used as the reference category. A *p*-value below 0.05 was considered significant.

## 3. Results

### 3.1. Sociodemographic Characteristics of the Studied Population

Table 1 below indicates that 59.8% (n = 447) of the 746 respondents were female. The younger age categories of 26–35 years old represented 28.8% (n = 215) of the population, followed by 18–25 years old representing 27.7% (n = 207). The racial breakdown of the sample was 29.9% (n = 223) white, 27.6% (n = 206) black, followed by coloured, Indian and Asian. The sample was dominated by residents living mainly in urban 65.8% (n = 491) and peri-urban areas 24.8% (n = 185) in the City of Johannesburg metropolitan 46.1% (n = 344), and the majority had tertiary education 61.9% (n = 462) as their highest level of education. A high number had a monthly income after tax ranging between ZAR 10,000-ZAR 14,999 (16.5%, n = 123) and ZAR 15,000-ZAR 24,999 (15.7%, n = 117). For international comparison, ZAR 1 = EUR 0.049 = USD 0.054.

### 3.2. Knowledge and Consumption Level of Indigenous Foods

Grain sorghum 32.4% (n = 242), marula 32% (n = 239), pearl millet 21.7% (n = 162), amadumbe 19.3% (n = 144) and cowpea 18.6% (n = 139) were the most known by respondents (Table 2). The top three consumed grain crops were grain sorghum 19.3% (n = 144), pearl millet 12.3% (n = 92) and cowpea 11.4% (n = 85). The top three consumed vegetable crops were amadumbe 12.3% (n = 92), amaranth 8.7% (n = 65) and cleome 8.3% (n = 62). The top three consumed fruits were marula 18.9% (n = 141), Kei apple 7.8% (n = 58) and mobola plum 6.7% (n = 50).

Table 3 illustrates the respondents’ consumption of the top three indigenous grain crops, vegetable crops and fruits, stratified by race. Globally, a significant relationship exists between these variables when considering Pearson chi-square values with *p* < 0.05, except for mobola plum. As shown in Table 3, the black population appeared to have a higher consumption compared to Asian, coloured, Indian and white people. Indeed, the odd ratios associated with this sub-population were generally significantly higher compared to those of the other races. As an example, the black population were sixteen times, nine times and three times more likely to consume amadumbe (OR: 16.17, 95%CI 6.33–41.32), pearl millet (OR: 9.48, 95%CI 4.56–19.71) and marula (OR: 3.13, 95%CI 1.92–5.10) than white people, respectively.

Those who consumed IF were further asked to indicate the frequency of the consumption (daily, weekly, monthly, seasonally or rarely). The consumption trends appeared more seasonal for most of these foods (Table 4).

The respondents did not consume much of the indigenous crops daily, weekly or monthly. For grains, Bambara groundnuts and cowpea were the most seasonally consumed (47.1% and 41.2% of consumers, respectively). Similarly, Jew’s mallow (51% of consumers), blackjack (44.3% of consumers) and amaranth (43.1% of consumers) were the most seasonally consumed for vegetables and with all fruits (25.6–43.8% of respondents). When asked which member of their families mostly consumed indigenous foods, 61% (n = 453) of the respondents stated that they do not consume indigenous foods but 23% (n = 169) mentioned that women and men equally consume indigenous foods in their households.

**Figure 1:**
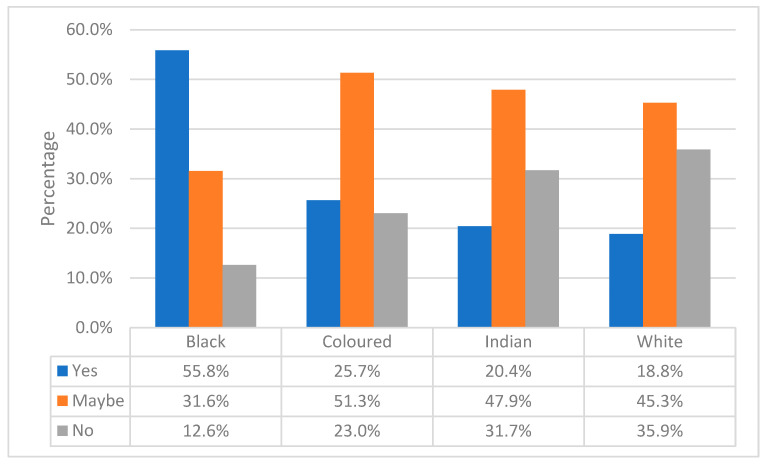
Desire to increase consumption of indigenous foods stratified by race. Gauteng province, South Africa, 2019.

Figure 1 shows the participants’ responses (stratified by race) concerning their willingness to consume indigenous foods more often. The majority stated “yes” or “maybe”.

### 3.3. Perceptions and Reasons for Consuming Indigenous Foods

**Figure 2:**
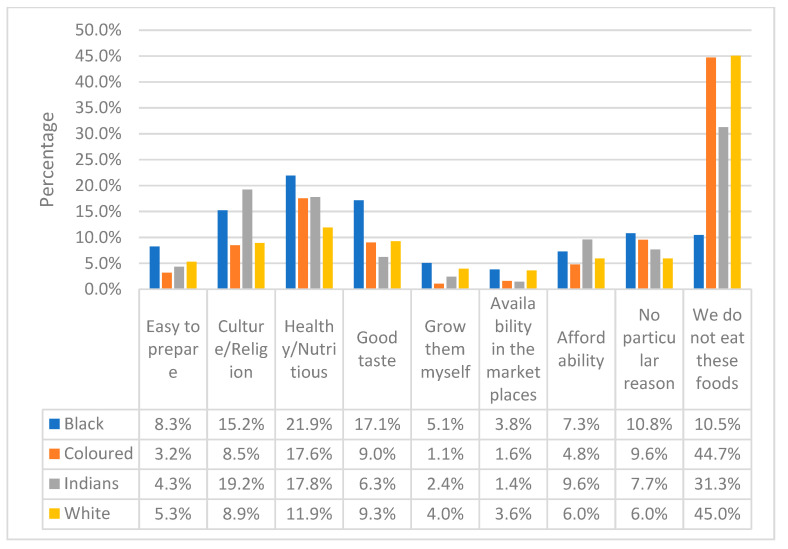
Respondents’ motivation for consuming indigenous foods stratified by race. Gauteng province, South Africa, 2019.

The results in Figure 2 illustrate the respondents’ motivations for consuming indigenous foods. The results are stratified by race. One of the common reasons for all races was “health and nutrition”. The highest responses were from the black respondents at 21.9%, followed by the coloured, Indian and white respondents at 17.6%, 17.8% and 11.9%, respectively. The Indian 19.2% and black 15.2% respondents indicated “culture/religion” as a reason, while black (17.1%) respondents also selected “good taste”.

**Figure 3:**
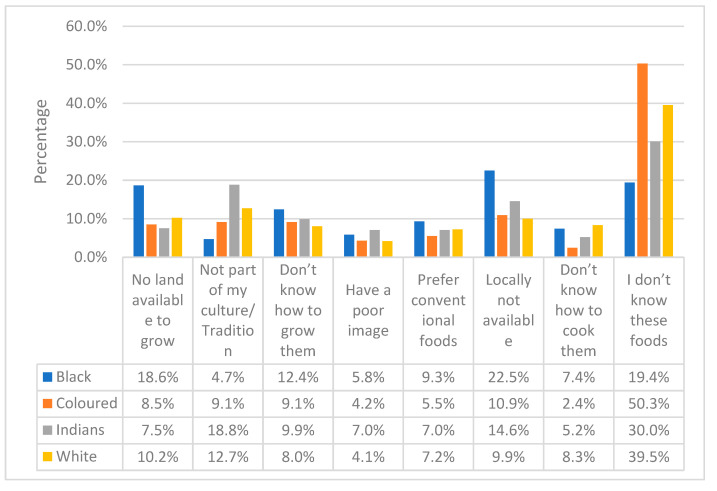
Respondents’ reasons for no or low consumption of indigenous foods stratified by race. Gauteng province, South Africa, 2019.

When the reason for no or low consumption of indigenous foods was requested (Figure 3), the respondents gave two common reasons: “no land available to grow” and “locally not available”. Availability seemed to be a problem contributing to low consumption. The black respondents scored (22.5%), followed by the coloured, Indian and white respondents at 10.9%, 14.6% and 9.9%, respectively, who mentioned that indigenous foods were “locally not available”. Interestingly, all races rated the reason “poor image” the lowest, with black at 5.8%, coloured at 4.2%, Indian at 7.0% and white at 4.1%. Indian (18.8%) and white (12.7%) respondents also mentioned that the low or non-consumption of IF is because such foods were not part of their culture.

## 4. Discussion

The population in this study was overall educated with a moderate household income per month after tax. The respondents answered the question of whether they knew the indigenous crops listed in the questionnaire by indicating either yes or no. Cultural differences emerged as more black respondents could identify more indigenous foods. Indigenous and traditional foods and dishes are passed down over generations or have been consumed for many generations [24,25]. Therefore, most of the foods in question could have constituted a larger part of the African diet since people in rural areas have relied on IFs for many years [26]. These populations can identify indigenous grains, vegetables and fruits; they know how to plant and harvest these crops and process the food [27]. This cultural aspect was mentioned by some Indians and whites as a reason for their low or nonconsumption of IFs.

Consumption data reveal that most indigenous foods are available seasonally. According to Mbhenyane [11], evidence from studies conducted in South Africa and around the world indicates that indigenous foods are consumed seasonally by many households, especially in areas where they are readily available. The majority of the respondents (65.8%) in the current study resided in urban areas where indigenous foods are not readily available and accessible; only 9.2% of the respondents resided in rural areas. According to Spires [28], urbanisation is commonly associated with the “nutrition transition”, giving rise to and accelerating profound shifts in diets, physical activity and the prevalence of the double burden of malnutrition. There has been an observed change in diet within the South African population, namely the transition from traditional/indigenous diets to Western diets. This confirms the statement by Nengovhela et al. [29] in a study conducted in 2018 in the Limpopo province that “Indigenous vegetables are popular in rural areas, there is evidence of studies that support these findings suggesting that indigenous vegetables are mostly grown in rural areas, supporting a significant proportion of households”. They further state that rural inhabitants can consume more indigenous foods because those are more widely available and accessible in rural areas than in urban environments. The respondents in this study did not consume much of the indigenous grain crops, vegetable crops or fruits daily, weekly or monthly, but most of them stated that they consumed the indigenous grain crops, vegetable crops and fruits seasonally, as expected. Ferguson et al. [30] conducted a study on traditional food availability and consumption in remote Aboriginal communities in the Northern Territory of Australia. They observed that traditional foods were consistently reported for all 20 communities to be available year-round but of different varieties based on season. In a study conducted in Botswana in 2020, Bultosa et al. [31] assert that 50 wild edible plants are used, most of which are available during the rainy season. Maize, sorghum, beans, cooking melon, watermelon and pumpkin are widely used domesticated crops in Botswana. Their further concern is that even though most traditional foods/dishes and beverages are still consumed and enjoyed by the communities in Botswana, the young generation has limited knowledge and skills on how to process and utilise them.

When asked which member of the family mostly consumed indigenous foods, a quarter (n = 169) mentioned that women and men consume indigenous foods equally in their households. Respondents were asked if they were willing to consume indigenous foods more often, and the responses were overall positive. As reported by Mbhenyane [11], in South Africa, the consumption pattern is highly variable and depends on geographic location, with the highest consumption mainly in rural areas. Many people consumed indigenous foods that they believed would reduce the risk of certain diseases. Indeed, this study shows that one of the common reasons for consuming indigenous foods by all races was for health and nutrition; 17% of the black respondents, followed by Indian, coloured and white respondents, stated that they consumed indigenous foods for medicinal purposes. This is supported by the study conducted by Van der Hoeven in 2013 [25], who found that the knowledge of medicinal use and food preparation was significantly present in the community living deep within the village, whereas communities closer to urban areas (though still in a rural community) stated that they were unfamiliar with the use of these indigenous foods.

The statistical analysis of the data confirms the relation between race and the consumption of indigenous foods (*p* < 0.05). The black population appeared more likely to consume indigenous grain, vegetable and fruit crops. The relation between race and the consumption of mobola plum was not significant; the consumption of this fruit was not as high as that of the other fruits. A possible reason for low consumption could be that some respondents did not know this fruit since only 10.2% of the respondents could identify it. According to DAFF [32], the mobola plum is a tropical tree native to Africa and is found in bushveld areas such as the Limpopo and Mpumalanga provinces.

The results show that a third of this study’s respondents do not consume indigenous foods. Those who did were motivated by health and nutrition, culture and religion and good taste; a very small percentage mentioned ease of preparation, affordability, growing such food themselves, their availability in the marketplace and no particular reason. Some of the respondents also mentioned that they did not know those foods. The two main reasons for low consumption were that the foods were not locally available and that no land was available to grow indigenous foods. A study by Macintyre et al. [33] found that low urban consumption was due to growth and spatial limitations. It was interesting to observe that the reason “poor image” was rated the lowest by all races. Accordingly, this finding contradicts Cloete and Idsardi [18], Demi [34], Van der Merwe et al. [35] and Majova [36], who report that indigenous foods are associated with poverty and seen as poor man’s food or old-fashioned, and that the younger generation has a negative attitude towards indigenous foods and are reluctant to learn more about them. Gewa et al. [37] state that the consumption of indigenous foods has decreased over the years. Indigenous foods are seen as old-fashioned because the older generation prepares such foods while the younger generation has access to a wide range of modern food. Urbanisation is integral to the decrease in consumption of indigenous foods [38,39]. Due to limited knowledge and decreased use, there is a knowledge gap between the variety of edible indigenous foods and those varieties being consumed [40]. Despite their exposure to a wide range of nutritious food, many urban residents seek modern, convenient foods from restaurants and fast-food outlets; consequently, the consumption of traditional foods has decreased in urban areas [41]. Technology use, the marketing of Western foods and, conversely, the lack of marketing of indigenous foods have also led to a decrease in the consumption of such foods.

Food diversity and indigenous food systems are well known to be useful in the fight against diet-related chronic diseases. IFs are a source of healthy foods or beverages, and can be used for developing value-added healthy dietary strategies to combat the prevalence of NCDs in communities experiencing chronic disease epidemics [42]. As attested by Chiekhyoussef and Embashu [43], consumers with a higher education and income status are more health-conscious and should be encouraged to follow a traditional indigenous diet instead of a Western diet. Weinberger [44] further states that selected indigenous foods are becoming more attractive to wealthier populations in East Africa and South-East Africa as these foods are moving into the commercial mainstream. Indeed, the nutritional and health value of IFs do not need to be demonstrated anymore. These grains, cereals, legumes, tubers, roots and fruits could be rich sources of macronutrients and micronutrients or bioactive compounds that modulate metabolic processes, with a positive effect on human health [24]. Takaidza [16] described the potential of each of the South African indigenous foods listed in this study. Examples are: pearl millet is a rich source of polyunsaturated fatty acids, minerals (Fe, Zn, Ca, Mg and P) and fibres, and has anti-diabetic properties; sorghum has revealed activities against diabetes, obesity, hypertension and cancer; and marula is recommended to combat high blood pressure.

This study helps to understand one of the reasons for the high proportion of diet-related diseases in South Africa, when considering the low consumption of indigenous foods observed in an urbanised region like the Gauteng province. Despite the low number of Asian participants, this research work might serve as a basis for intervention strategies in the Gauteng region since Asians are a minority, yet the research could still help to predict populations’ behaviour in other urbanised regions of the country. However, gathering more data from populations residing in rural and peri-urban areas would be significant.

## 5. Conclusions

The findings of this study suggest little knowledge and consumption of indigenous foods by populations residing in the Gauteng region. Being black is associated with a higher consumption compared to being coloured, Indian or white. Black people also know, consume and have the ability to obtain indigenous foods from informal markets. There is a need to increase the accessibility and availability of those foods that appear to be consumed more seasonally to date and to enhance sensitisation about their nutritional and health value. The respondents suggested that indigenous foods should be more accessible and available in various supermarkets, schools and hospitals. Nutrition education and workshops about indigenous foods should be offered to consumers. These foods should be incorporated into hospital menus and feeding schemes at schools or within homes in the community, which would increase knowledge levels about indigenous foods as well as their consumption. Such steps would assist in combatting malnutrition and micronutrient deficiencies within the region and across the country. The IFs should be promoted because of their high phytochemical content and potential to prevent NCDs.

## Figures and Tables

**Table 1:** Demographic profile of respondents (n= 746).

Demographic Variables		Frequency (n)	Percentage (%)
Gender	Male	299	40.1
	Female	447	59.9
Race	Black	206	27.6
	Coloured	152	20.4
	Indian	142	19
	White	223	29.9
	Asian	21	2.8
	Other	2	0.3
Age group	18–25	207	27.7
	26–35	215	28.8
	36–45	171	22.9
	46–55	101	13.5
	56–65	37	5
	66+	14	1.9
	Missing	1	0.1
Highest education level	Grade 0–7	17	2.3
	Grade 8–12	267	35.8
	Tertiary Education	462	61.9
Household size	1–2	145	19.4
	3–5	441	59.1
	6 or more	160	21.4
Household Income per month after tax	Less than ZAR 500	16	2.1
	ZAR 500–ZAR 999	13	1.7
	ZAR 1000–ZAR 1999	20	2.7
	ZAR 2000–ZAR 2999	39	5.2
	ZAR 3000–ZAR 4999	84	11.3
	ZAR 5000–ZAR 9999	94	12.6
	ZAR 10,000–ZAR 14,999	123	16.5
	ZAR 15,000–ZAR 24,999	117	15.7
	ZAR 25,000–ZAR 34,999	82	11
	ZAR 35,000–ZAR 44,999	67	9
	ZAR 45,000–ZAR 54,999	47	6.3
	ZAR 55,000+	44	5.9
Area of residence	Urban	491	65.8
	Peri-urban	185	24.8
	Rural	69	9.2
	Missing	1	0.1
Corresponding settlement of urban/peri-urban area	Informal settlement	60	8
	Former border or homeland towns	11	1.5
	Township (Kasi)	189	25.3
	Suburb/Edge city	416	55.8
	Missing	70	9.4

**Table 2:** Frequency of knowledge and consumption of indigenous foods by the studied population (N = 746). Gauteng province, South Africa, 2019.

Indigenous Food		Knowledge n (%)	Consumption n (%)
Grain crops	Pearl millet	162 (21.7%)	92 (12.3%)
	Grain sorghum	242 (32.4%)	144 (19.3%)
	Cowpea	139 (18.6%)	85 (11.4%)
	Bambara groundnuts	112 (15.0%)	70 (9.4%)
	Mungbean	101 (13.5%)	60 (8.0%)
Vegetable crops	Cleome	102 (13.7%)	62 (8.3%)
	Amaranth	108 (14.5%)	65 (8.7%)
	Blackjack	126 (16.9%)	61 (8.2%)
	Jews mallow	74 (9.9%)	49 (6.6%)
	Cassava	98 (13.1%)	51 (6.8%)
	Amadumbe	144 (19.3%)	92 (12.3%)
Fruits	Marula	239 (32.0%)	141 (18.9%)
	Red milkwood	58 (7.8%)	39 (5.2%)
	Mobola plum	76 (10.2%)	50 (6.7%)
	Wild medlar	61 (8.2%)	43 (5.8%)
	Num-num	55 (7.4%)	32 (4.3%)
	Kei apple	90 (12.1%)	58 (7.8%)
	Natal orange (*Strychnos spinosa)*	88 (11.8%)	50 (6.7%)

**Table 3:** Frequency of consumption of indigenous foods, stratified by race, and associated odds ratio. Gauteng province, South Africa, 2019.

Race and consumption of indigenous grain crops									
Race	Pearl Millet			Grain Sorghum			Cowpea		
	n (%)	Odd ratio (95%CI)	*p*-Value	n (%)	Odd ratio (95%CI)	*p*-Value	n (%)	Odd ratio (95%CI)	*p*-Value
Asian	2 (9.5%)	2.5 (0.5–12.4)	0.262	3 (14.3%)	1.79 (0.48–8.63)	0.384	1 (4.8%)	0.96 (0.12–7.85)	0.972
Black	59 (28.5%)	9.48 (4.56–19.71)	0.000	86 (41.5%)	7.63 (4.42–13.16)	0.000	59 (28.5%)	7.68 (3.90–15.12)	0.000
Coloured	10 (6.5%)	1.65 (0.65–4.16)	0.288	24 (15.6%)	1.98 (1.04–3.76)	0.036	10 (6.5%)	1.34 (0.55–3.23)	0.517
Indian	13 (9.2%)	2.40 (0.99–5.76)	0.051	13 (9.2%)	1.08 (0.52–2.27)	0.834	5 (3.5%)	0.70 (0.24–2.07)	0.523
White	9 (4.0%)	1		19 (8.5%)	1		11 (4.9%)	1	
**Race and consumption of indigenous vegetable crops**									
**Race**	**Cleome**			**Amaranth**			**Amadumbe**		
	**n (%)**	**Odd ratio (95%CI)**	***p*-value**	**n (%)**	**Odd ratio (95%CI)**	***p*-value**	**n (%)**	**Odd ratio (95%CI)**	***p*-value**
Asian	0 (0%)	1.62 × 10^−8^ (1.62 × 10^−8^–1.62 × 10^−8^)	-	0 (0%)	2.01 × 10^−8^ (2.01 × 10^−8^–2.01 × 10^−8^)	-	0 (0%)	1.03 × 10^−8^ (1.03 × 10^−8^–1.03 × 10^−8^)	-
Black	42 (20.3%)	9.21 (3.82–22.17)	0.000	48 (23.2%)	13.16 (5.12–33.81)	0.000	56 (27.1%)	16.17 (6.33–41.32)	0.000
Coloured	5 (3.2%)	1.21 (0.36–4.05)	0.753	6 (3.9%)	1.77 (0.53–5.90)	0.354	13 (8.4%)	4.02 (1.40–11.52)	0.010
Indian	10 (7.0%)	2.74 (0.97–7.71)	0.056	6 (4.2%)	1.92 (0.58–6.43)	0.288	19 (13.4%)	6.74 (2.45–18.48)	0.000
White	(2.7%)	1		5 (2.2%)	1		5 (2.2%)	1	
**Race and consumption of indigenous fruit crops**									
**Race**	**Marula**			**Mobola plum**			**Kei apple**		
	**n (%)**	**Odd ratio (95%CI)**		**n (%)**	**Odd ratio (95%CI)**	***p*-value**	**n (%)**	**Odd ratio (95%CI)**	***p*-value**
Asian	1 (4.8%)	0.33 (0.04–2.59)	0.294	0 (0%)	5.81 × 10^−9^ (5.81 × 10^−9^–5.81 × 10^−9^)	-	1 (4.8%)	0.96 (0.12–7.85)	0.972
Black	66 (31.9%)	3.13 (1.92–5.10)	0.000	22 (10.6%)	1.92 (0.94–3.92)	0.073	30 (14.5%)	3.26 (1.59–6.70)	0.001
Coloured	26 (16.9%)	1.36 (0.76–2.41)	0.295	7 (4.5%)	0.77 (0.30–1.98)	0.585	7 (4.5%)	0.92 (0.35–2.42)	0.862
Indian	20 (14.1%)	1.10 (0.59–2.02)	0.768	8 (5.6%)	0.96 (0.39–2.39)	0.938	9 (6.3%)	1.30 (0.53–3.23)	0.566
White	29 (13.0%)	1		13 (5.8%)	1		11 (4.9%)	1	

**Table 4:** Consumption frequencies of indigenous foods by respondents. Gauteng province, South Africa, 2019.

Indigenous Foods		Frequency					Missing n (%)	Total n (%)
		Daily n (%)	Weekly n (%)	Monthly n (%)	Seasonally n (%)	Rarely n (%)		
Grain crops	Pearl millet	9 (9.8%)	19 (20.7%)	18 (19.6%)	20 (21.7%)	22 (23.9%)	4 (4.3%)	92 (100%)
	Grain sorghum	12 (8.3%)	28 (19.4%)	20 (13.9%)	33 (22.9%)	42 (29.2%)	9 (6.3%)	144 (100%)
	Cowpea	1 (1.2%)	6 (7.1%)	19 (22.4%)	35 (41.2%)	15 (17.6%)	9 (10.6%)	85 (100%)
	Bambara groundnuts	2 (2.9%)	3 (4.3%)	4 (5.7%)	33 (47.1%)	18 (25.7%)	10 (14.3%)	70 (100%)
	Mungbean	1 (1.7%)	11 (18.3%)	8 (13.3%)	15 (25%)	20 (33.3%)	5 (8.3%)	60 (100%)
Vegetable crops	Cleome	4 (6.5%)	8 (12.9%)	6 (9.7%)	23 (37.1%)	17 (27.4%)	4 (6.5%)	62 (100%)
	Amaranth	1 (1.5%)	5 (7.7%)	6 (9.2%)	28 (43.1%)	19 (29.2%)	6 (9.2%)	65 (100%)
	Blackjack	2 (3.3%)	4 (6.6%)	6 (9.8%)	27 (44.3%)	15 (24.6%)	7 (11.5%)	61 (100%)
	Jew’s mallow	1 (2%)	5 (10.2%)	4 (8.2%)	25 (51%)	11 (22.4%)	3 (6.1%)	49 (100%)
	Cassava	1 (2%)	3 (5.9%)	5 (9.8%)	19 (37.3%)	14 (27.5%)	9 (17.6%)	51 (100%)
	Amadumbe	2 (2.2%)	4 (4.3%)	17 (18.5%)	34 (37%)	27 (29.3%)	8 (8.7%)	92 (100%)
Fruits	Marula	4 (2.8%)	4 (2.8%)	2 (1.4%)	59 (41.8%)	59 (41.8%)	13 (9.2%)	141 (100%)
	Red milkwood	1 (2.6%)	6 (15.4%)	3 (7.7%)	14 (35.9%)	11 (28.2%)	4 (10.3%)	39 (100%)
	Mobola plum	2 (4%)	1 (2%)	3 (6%)	21 (42%)	15 (30%)	8 (16%)	50 (100%)
	Wild medlar	1 (2.3%)	4 (9.3%)	1 (2.3%)	11 (25.6%)	17 (39.5%)	9 (20.9%)	43 (100%)
	Num-num	0 (0%)	0 (0%)	6 (18.8%)	14 (43.8%)	8 (25%)	4 (12.5%)	32 (100%)
	Kei apple	4 (6.9%)	2 (3.4%)	4 (6.9%)	27 (46.6%)	16 (27.6%)	5 (8.6%)	58 (100%)
	Natal plum	3 (6%)	2 (4%)	5 (10%)	19 (38%)	15 (30%)	6 (12%)	50 (100%)

## Data Availability

Data available on request due to restrictions, e.g., privacy or ethical.
